# Supplementary material for: AGEs and sRAGE Variations at Different Timepoints in Patients with Chronic Kidney Disease
Source: Antioxidants (Basel). 2021 Dec 15;10(12):1994. doi: 10.3390/antiox10121994 (PMC8698924; doi:10.3390/antiox10121994)
Supplement: Supplementary file 1 [file antioxidants-10-01994-s001.zip › antioxidants-1464923-supplementary.pdf]

Supplementary Materials

**Table S1** harmacologic treatment at baseline (BL) and follow-up (FU)

| Pharmacologic agents | BL n (%) | FU n (%) |
|---|---|---|
| RAS inhibitors | 34 (53) | 27 (42) |
| Diuretics | 29 (45) | 35 (55) |
| Statins | 46 (72) | 49 (76) |
| Bicarbonate | 24 (38) | 28 (44) |
| Allopurinol | 50 (78) | 47 (73) |
| Hypoglycaemic agents | 24 (37) | 26 (41) |

RAS, renin-angiotensin system.

**Table S2** Metabolic and renal function parameters of recruitable patients and deceased/dialyzed at baseline

| Variables | Recruitable patients (n=98) | Dialysis/Death (n=34) | p |
|---|---|---|---|
| eGFR, (ml/min) | 27±10 | 17±8 | **<0.0001** |
| Age, (years) | 80 [74;84] | 79 [74;85] | 0.78 |
| BMI (kg/m$^2$) | 28.4±4.8 | 26.1±3.9 | 0.61 |
| Fasting blood Glucose (mg/dl) | 104 [93;139] | 101 [86;131] | 0.16 |
| Albumin (g/dl) | 4.0±0.3 | 4±0.4 | 0.11 |
| Uric Acid (mg/dl) | 6.0±1,3 | 6.5±1.6 | 0.31 |
| Total Cholesterol (mg/dl) | 165±30 | 176±48 | 0.09 |
| HDL (mg/dl) | 48 [40;60] | 54 [44;66] | 0.12 |
| LDL (mg/dl) | 84 [68;102] | 87 [67;116] | 0.49 |
| Triglycerides (mg/dl) | 117 [94;153] | 111 [87;150] | 0.21 |
| HbA1c (mmol/dl) | 48.5±11 | 43±10 | **0.05** |
| Proteinuria (mg/24h) | 456 [206;1090] | 977 [204;3073] | 0.10 |
| Systolic Pressure (mmHg) | 135 [125;144] | 130 [110;148] | 0.22 |
| Diastolic Pressure (mmHg) | 80 [70;89] | 90 [66;110] | 0.31 |

eGFR: estimated glomerular filtration rate; HbA1c: glycated hemoglobin; HDL: high density lipoprotein; LDL: Low density lipoprotein.
